# Association between the Degree of Pre-Synaptic Dopaminergic Pathway Degeneration and Motor Unit Firing Behavior in Parkinson’s Disease Patients

**DOI:** 10.3390/s21196615

**Published:** 2021-10-04

**Authors:** Yuichi Nishikawa, Kohei Watanabe, Aleš Holobar, Tetsuya Takahashi, Noriaki Maeda, Hirofumi Maruyama, Shinobu Tanaka, Allison S Hyngstrom

**Affiliations:** 1 Faculty of Frontier Engineering, Institute of Science & Engineering, Kanazawa University, Kanazawa 920-1192, Japan; shinobu@se.kanazawa-u.ac.jp; 2 Laboratory of Neuromuscular Biomechanics, School of Health and Sport Sciences, Chukyo University, Toyota 470-0393, Japan; wkohei@lets.chukyo-u.ac.jp; 3 Faculty of Electrical Engineering and Computer Science, University of Maribor, 2000 Maribor, Slovenia; ales.holobar@um.si; 4 Department of Rehabilitation, Faculty of Rehabilitation, Hiroshima International University, Higashihiroshima 739-2695, Japan; tetakaha@me.com; 5 Division of Sports Rehabilitation, Graduate School of Biomedical and Health Sciences, Hiroshima University, Hiroshima 734-8553, Japan; norimmi@hiroshima-u.ac.jp; 6 Department of Clinical Neuroscience and Therapeutics, Graduate School of Biomedical and Health Sciences, Hiroshima University, Hiroshima 734-8551, Japan; hmaru@hiroshima-u.ac.jp; 7 Department of Physical Therapy, Marquette University, Milwaukee, WI 53201, USA; Allison.hyngstrom@marquette.edu

**Keywords:** EMG recording, Parkinson’s disease, motor unit recruitment, dopamine receptor

## Abstract

The relationship between motor unit (MU) firing behavior and the severity of neurodegeneration in Parkinson’s disease (PD) is not clear. This study aimed to elucidate the association between degeneration with dopaminergic pathways and MU firing behavior in people with PD. Fourteen females with PD (age, 72.6 ± 7.2 years, disease duration, 3.5 ± 2.1 years) were enrolled in this study. All participants performed a submaximal, isometric knee extension ramp-up contraction from 0% to 80% of their maximal voluntary contraction strength. We used high-density surface electromyography with 64 electrodes to record the muscle activity of the vastus lateralis muscle and decomposed the signals with the convolution kernel compensation technique to extract the signals of individual MUs. We calculated the degree of degeneration of the central lesion-specific binding ratio by dopamine transporter single-photon emission computed tomography. The primary, novel results were as follows: (1) moderate-to-strong correlations were observed between the degree of degeneration of the central lesion and MU firing behavior; (2) a moderate correlation was observed between clinical measures of disease severity and MU firing behavior; and (3) the methods of predicting central nervous system degeneration from MU firing behavior abnormalities had a high detection accuracy with an area under the curve >0.83. These findings suggest that abnormalities in MU activity can be used to predict central nervous system degeneration following PD.

## 1. Introduction

Parkinson’s disease (PD) is a common neurodegenerative disorder that affects approximately 1% of people older than 60 years [1]. Motor dysfunction in people with PD is caused by a disturbance of the central nervous system where the degeneration of dopaminergic pathways within the basal ganglia results in impaired muscle contraction [2]. It has been reported that neurodegeneration begins in people with PD many years before the onset of physical symptoms, and that prodromal symptoms, such as constipation, dizziness, and sleep disturbances are frequently observed at least 10 years before disease onset [3]. These symptoms suggest that the decrease in the number of neurons in people with PD begins at an early stage, and it is important to accurately diagnose the signs of PD during the prodromal phase and to begin therapeutic intervention at an early stage. Large-scale studies have been conducted on diagnostic biomarkers of PD and indicators using cerebrospinal fluid and other data have been reported [4], but diagnostic biomarkers using noninvasive methods have not yet been established.

Recently, noninvasive methods (high-density surface electromyography (HD-SEMG)) of analyzing motor units (MUs) have been developed [5,6,7,8], and research has been conducted on abnormalities in MU firing behavior in people with PD [9,10,11]. A previous study reported a correlation between clinical measures of the severity of PD and MU firing behavior [11], and it is plausible that this correlation can be used to evaluate disease severity. However, no studies have examined the association of the degree of neurodegeneration and abnormalities in MU firing behavior in people with PD. Previous studies suggest that MU firing behavior could be used to predict central nervous system degeneration. Our previous study showed that abnormal MU firing behavior can be detected even in people with mild symptoms (disease duration = 2.7 ± 0.9 years, Unified Parkinson’s disease rating scale (UPDRS) Part III = 13 (9–16), median (min–max)) with respect to MU firing behavior in healthy subjects [11]. Notably, even though the people with PD in the present study were independent in their activities of daily living, the HD-SEMG method was able to detect clear differences between PD and healthy subjects [11]. Thus, the use of HD-SEMG may be a good candidate for early PD detection. Dopamine transporter single photon emission computed tomography (DAT-SPECT) is often used to indirectly assess the degree degeneration of the central nervous system in people with PD [12]. However, due to the effects of radiation exposure and other factors, DAT-SPECT is not regularly performed. If possible, the prediction of central nervous system degeneration based on abnormalities in MU firing behavior could be useful for the early diagnosis of PD.

Therefore, the purpose of this study was to elucidate the association of the severity of central nervous system degeneration and abnormalities in MU firing behavior in people with PD (including those with mild-to-moderate symptoms). We hypothesized that a correlation exists between the extent of degeneration of the central nervous system and MU firing behavior abnormalities and that the analysis of MU firing behavior can be an effective tool for the assessment of neurodegeneration.

## 2. Materials and Methods

### 2.1. Subjects

Fourteen females with PD were enrolled in this study (age, 72.6 ± 7.2 years, disease duration, 3.5 ± 2.1 years). There is a sex difference in the physical symptoms of PD [13,14], and a previous study reported that females exhibited more asymmetry of motor function than males in PD [15]. Therefore, to exclude any influence of sex on physical symptoms and MU firing behavior, only females were included in this study. The inclusion criteria were as follows: female sex, Hoehn and Yahr stage < 4, and Parkinson’s disease. The exclusion criteria were as follows: taking selective serotonin reuptake inhibitors or antidepressant medication, and neurodegenerative diseases other than PD (e.g., Alzheimer’s disease, frontotemporal dementia, progressive supranuclear palsy, and dystonia). The UPDRS Part III was used to assess motor symptoms. The same neurologist (T.T.) performed the UPDRS Part III assessments on all participants. The more-affected side was determined by the participant’s medical history.

### 2.2. Muscle Strength Testing and Physical Symptom Assessment

The participants underwent maximal voluntary isometric contraction (MVC) of knee extension muscle strength in each leg, and the order of measurement was randomized. The BIODEX system (BIODEX System 4; Biodex Medical Systems, Shirley, NY, USA) was used to measure MVC. The hip and knee joint were maintained at 90° during MVC measurements (180° indicates full extension). The participants performed two MVC trials, with a 10-min warm-up (e.g., stretching) before the MVC measurement and at least 2 min of rest between trials [16]. The higher torque was adopted as the MVC value. The participants performed a submaximal ramp-up contraction task of knee extensors up to 80% MVC (ramp-up rate was 10% MVC/sec) after the MVC measurements. The participant’s torque and target torque during the ramp-up contraction task were projected on a monitor.

### 2.3. Electromyography (EMG) Recording

**Figure 1:**
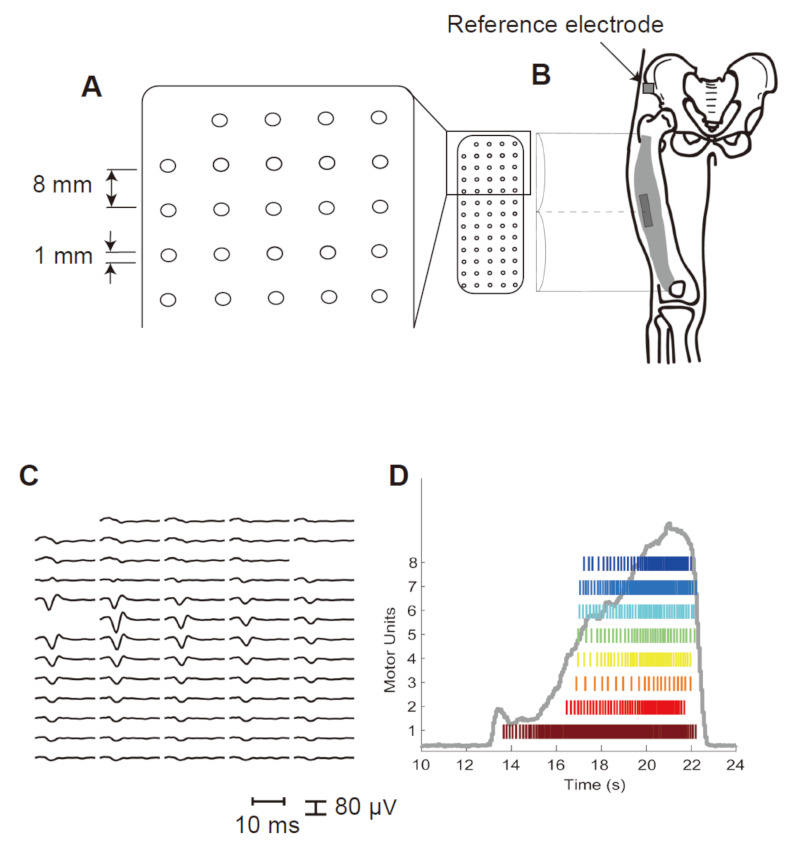
(**A**) The grid consisted of 13 rows and 5 columns of electrodes (1 mm diameter, 8 mm interelectrode distance in each direction), with one missing electrode in the upper left corner. (**B**) The high-density surface electromyography (HD-SEMG) electrode grid was placed on the vastus lateralis muscle. Multiple-electrodes were attached at the center of the line between the femoral greater trochanter protuberance and the lateral edge of the patella. A reference electrode was attached at the anterior superior iliac spine. (**C**) Motor unit (MU) action potential templates identified by the convolution kernel compensation technique from the HD-SEMG signals. Blanks in the MU template are discarded SEMG channels. (**D**) Person with Parkinson’s disease raster plot of incidences of action potentials superimposed on the torque generated during ramp-up contraction.

HD-SEMG signals were detected with a semi-disposable grid of 64 electrodes (ELSCH064NM2, OT Bioelettronica, Torino, Italy, 13 rows and 5 columns; 1 mm diameter, 8 mm interelectrode distance in each direction) during MVC and submaximal ramp-up contraction tasks according to the same procedure used in previous studies [16,17,18,19]. (Figure 1A). The participant’s skin was prepared by removing the hair and cleaning with alcohol. Multiple-electrodes were attached at the center of the line between the femoral greater trochanter protuberance and the lateral edge of the patella using a biadhesive sheet (KITAD064, OT Bioelettronica) with conductive paste (Elefix Z-181BE, NIHONKOHDEN, Tokyo, Japan). A reference electrode was attached at the anterior superior iliac spine [17] (Figure 1B). Monopolar HD-EMG signals (64 channels) were recorded with an off-line bandpass filter (10–500 Hz), amplified by a factor of 1000 with a sampling frequency at 2048 Hz and digitized by a 12-bit A/D converter (EMG-USB2+, OTBioelettronica).

### 2.4. Data Processing

The recorded monopolar signals were transferred to analysis software (MATLAB 2020b, MathWorks GK, MA, USA). The individual MUs were identified by the convolution kernel compensation (CKC) technique using DEMUSE software (Figure 1C,D) [7,8,20]. As a measure of MU identification accuracy, we used the pulse-to-noise ratio introduced by Holobar [21], and only MU signals with a pulse-to-noise ratio of 30 dB or higher (corresponding to MU firing identification accuracy of 90% or higher) were used for further analysis, while other MU signals were discarded [21]. After MU decomposition, the discharge patterns of individual MUs were inspected and evaluated by one investigator (Y.N.). The method used to manually edit the decomposition results is as follows. (1) The spike train of the MU was checked and edited throughout the entire contraction. (2) Unreliable MUs with a pulse-to-noise ratio of less than 30dB were removed. (3) The discharge times of individual MUs were used to calculate the instantaneous MU firing rate. (4) Abnormal interspike intervals (<33.3 ms or >250 ms) were excluded from the calculation [7,22,23]. The mean and coefficient of variation (CV) of the MU firing rate of individual MUs were calculated from the average and standard deviation (SD) of the instantaneous firing rates during the ramp-up contraction task. We used only < 30% CV for further analysis [24]. The range of MU firing rates was 4.3–19.3 pps for the more-affected side and 4.2–16.8 pps for the less-affected side. For each participant, the linear regression slope between the mean MU firing rate and the recruitment threshold was calculated.

### 2.5. DAT-SPECT

PECT images were obtained with ioflupane, an iodine-labeled ligand, with ^123^I-FP-CIT. Ioflupane was administered intravenously, and a scintigram of the head was obtained 3–6 h after administration using a SPECT-CT device (BRIGHTVIEW X, Philips Healthcare, Best, the Netherlands). A nuclear medicine physician blinded to the participants’ clinical information measured the specific binding ratio (SBR) using DaTView^®^ (Nihon Medi-Physics, Tokyo, Japan). The image reconstruction condition of DaTView^®^ was the filtered back projection method based on the report by Bolt et al. [25]. A Butterworth filter with a cutoff frequency of 0.45 cycles/cm was used as the preprocessing filter, and attenuation correction was performed with the Chang method (linear attenuation coefficient: 0.07 cm^−1^). DAT-SPECT was performed during the off-medication period. All participants underwent DAT-SPECT scans within one month before and after the EMG measurements.

### 2.6. Statistical Analysis

Before the analysis, the normal distribution of the data was confirmed using the Shapiro–Wilk test. The laterality of the MVC value, CV of the mean MU firing rate, recruitment threshold, and SBR_Bolt_ were analyzed by paired *t*-tests. Associations between the UPDRS Part III scores and SBR_Bolt_ and between the SBR_Bolt_ and mean MU firing rate, CV of the mean MU firing rate, cross-correlation function, and linear regression slope were analyzed by using Pearson correlation coefficients. The correlation coefficients were qualitatively interpreted using the following threshold according to a previous study: 0.2–0.4 indicates small; 0.4–0.7 indicates moderate; 0.7–0.9 indicates strong; and 0.9–1.0 indicates very strong [11]. Tossici-Bolt et al. reported that a cutoff SBR of ~ 4.5 distinguished normal and abnormal groups with a sensitivity, specificity, and diagnostic concordance of 97%, 92%, and 95%, respectively [25]. We used cutoff values from their study as a reference and to determine the cutoff mean MU firing rate, CV of the mean MU firing rate, cross-correlation function, and linear regression slope for predicting the severity of degeneration of lesions (SBR > 4.5 or SBR < 4.5), and receiver operating characteristic (ROC) curves were constructed. The cutoff value was calculated by Youden’s index. Statistical analyses were performed using SPSS version 25.0 (SPSS, Inc., Chicago, IL, USA). The level of significance was set at 0.05. We used Bonferroni correction for the Pearson correlation coefficients (critical alpha < 0.05/4 = 0.013).

## 3. Results

The characteristics of the participants are shown in Table 1. The knee extension torque was significantly lower on the more-affected side than on the less-affected side (*p* < 0.001).

**Figure 2:**
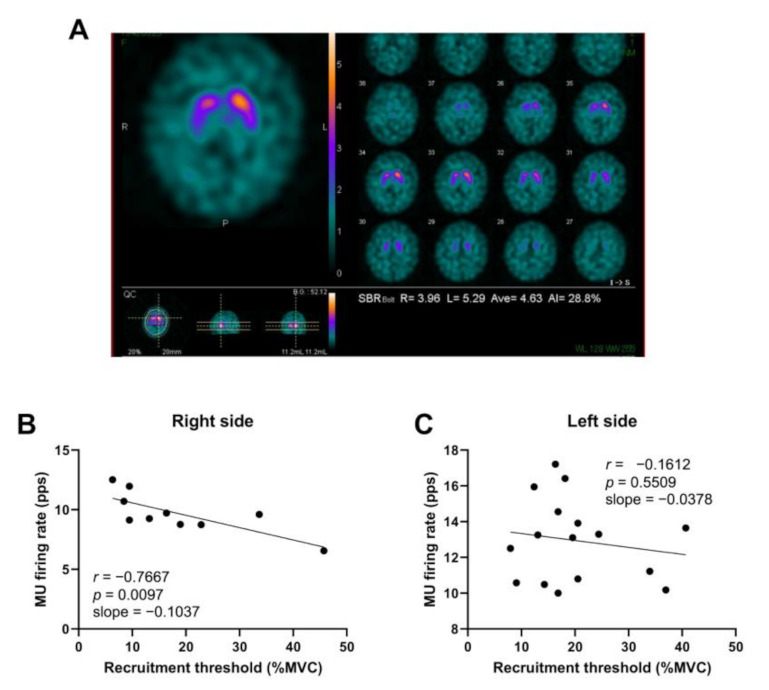
Representative data of DAT-SPECT (**A**) and MU firing behavior (**B**,**C**) of a female with Parkinson’s disease (71 years old, UPDRS Part III score = 8) during the ramp-up contraction task. Neurodegeneration can be seen predominantly on the right side. Regarding the MU firing behavior the correlation between the firing rate and recruitment threshold showed a negative correlation on the right side, while no correlation was observed on the left side.

Figure 2 shows representative data of DAT-SPECT and MU firing behavior during the ramp-up contraction task in a female with PD. People with PD present with motor symptoms on the contralateral side of the neurodegenerative process. The right side (less-affected side) showed a negative correlation between the MU firing rate and recruitment threshold, while the left side (more-affected side) showed no correlation.

**Figure 3:**
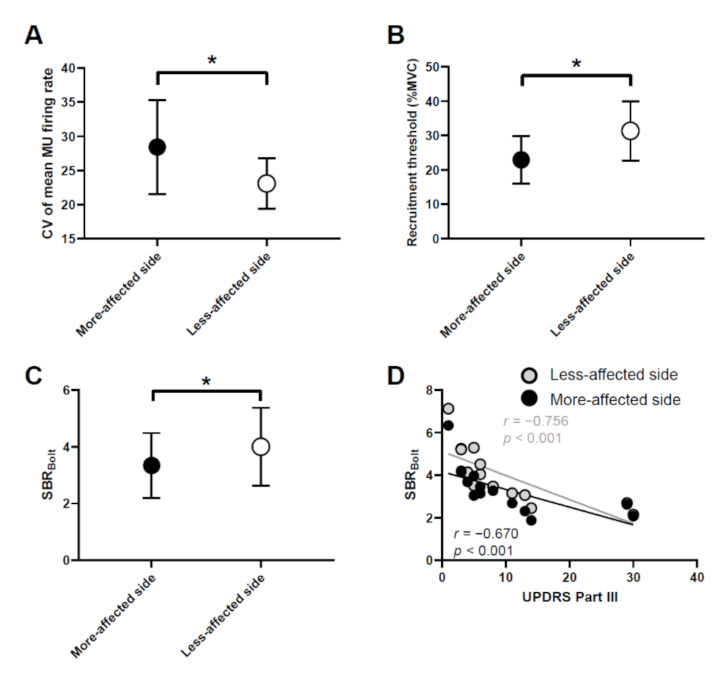
Comparison of the coefficient of variation of the motor unit (MU) firing rate (**A**), recruitment threshold (**B**), and SBR_Bolt_ (**C**) on the more- and less-affected sides and the relationship between the UPDRS Part III and SBR_Bolt_ (**D**) in people with Parkinson’s disease. * *p* < 0.05.

The more-affected side showed a significantly higher CV of the mean MU firing rate and a significantly lower recruitment threshold and SBR_Bolt_ than the less-affected side (*p* < 0.001, *p* = 0.005, and *p* < 0.001, respectively, Figure 2A–C). Moderate to very strong correlations were observed between the UPDRS Part III scores and SBR_Bolt_ on both sides in people with PD (more-affected side: *r* = −0.670, *p* < 0.001; less-affected side: *r* = −0.756, *p* < 0.001, Figure 3D). 

**Figure 4:**
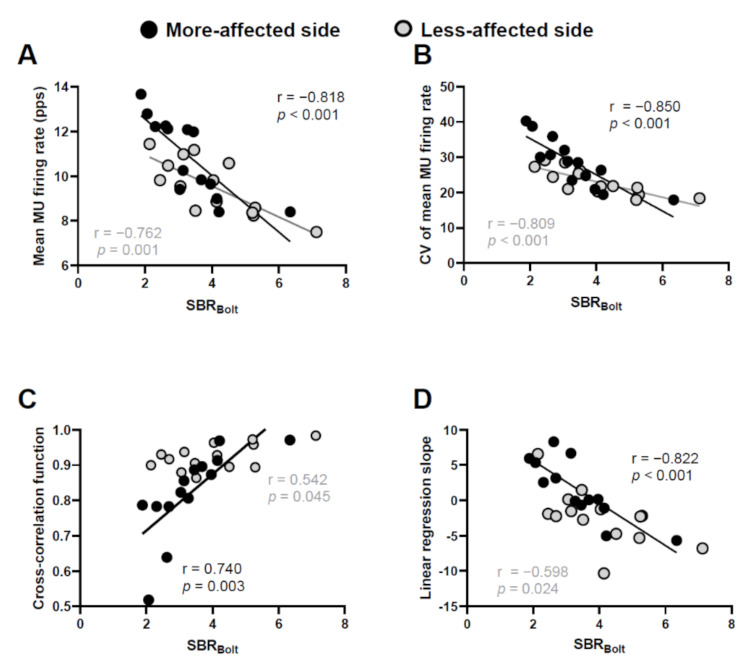
Relationship between the SBR_Bolt_ and mean motor unit (MU) firing rate (**A**), coefficient of variation (CV) of the mean MU firing rate (**B**), cross-correlation function (**C**), and linear regression slope (**D**). *p* < 0.013 (Bonferroni correction, *p* = 0.05/4).

Strong correlations were observed between the SBR_Bolt_ and mean MU firing rate, CV of the mean MU firing rate, cross-correlation function, and linear regression slope on the more-affected side (r = −0.818, *p* < 0.001, r = −0.850, *p* < 0.001, r = 0.740, *p* = 0.003, and r = −0.822, *p* < 0.001, respectively, Figure 4). The less-affected side showed moderate correlations between the SBR_Bolt_ and the mean MU firing rate and CV of the mean MU firing rate (r = −0.7621, *p* = 0.001, and r = −0.809, *p* < 0.001, respectively, Figure 4A,B).

**Figure 5:**
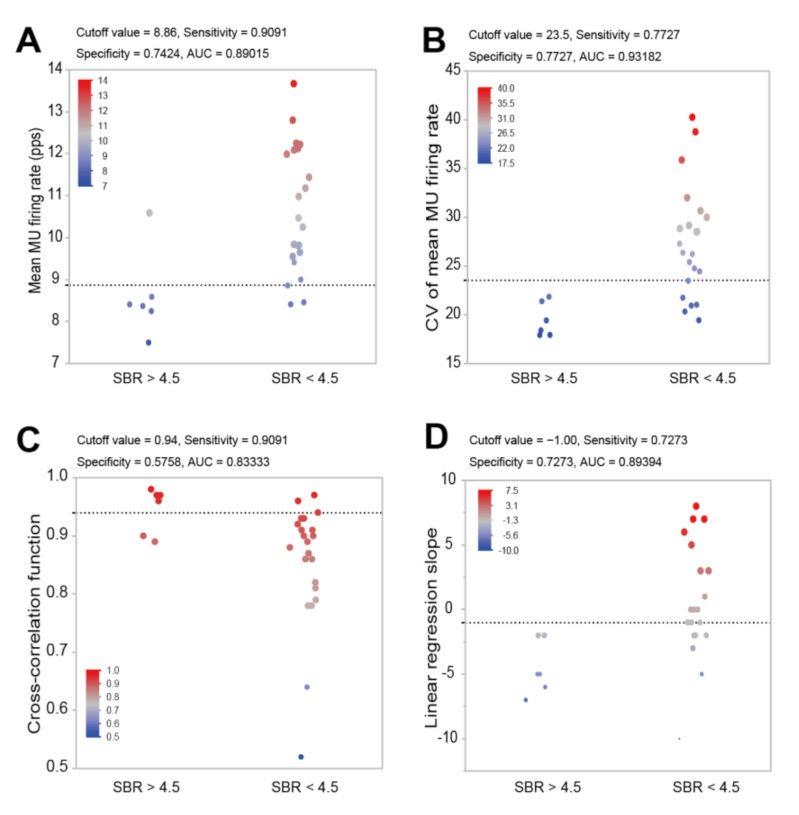
Cutoff values for the mean motor unit (MU) firing rate (**A**), coefficient of variation (CV) of the mean MU firing rate (**B**), cross-correlation function (**C**), and linear regression slope (**D**) that predict degeneration of the lesion.

The cutoff values for the mean MU firing rate, CV of the mean MU firing rate, cross-correlation function, and linear regression slope that predict degeneration of the lesion are shown in Figure 5. The mean MU firing rate cutoff value was 8.86 pps (sensitivity = 0.9091, specificity = 0.7424, and area under the curve (AUC) = 0.89015), the CV of the mean MU firing rate was 23.5 (sensitivity = 0.7727, specificity = 0.7727, AUC = 0.93182), the cross-correlation function was 0.94 (sensitivity = 0.9091, specificity = 0.5758, and AUC = 0.83333), and the linear regression slope was −1.00 (sensitivity = 0.7273, specificity = 0.7273, and AUC = 0.89394).

## 4. Discussion

This study compared the severity of degeneration of central lesions and MU firing behavior in people with PD. The primary novel results were that moderate to strong correlations were observed between the degeneration of the central lesion and MU firing behavior and disease severity. These findings support our hypothesis that MU firing behavior is related to the degree of central lesion degeneration. Furthermore, MU firing behavior may predict the severity of central lesion degeneration.

The more-affected side showed a lower knee extensor torque, recruitment threshold, and SBR_Bolt_, and a higher CV of the mean MU firing rate than the less-affected side. Previous studies have reported that people with PD exhibit laterality in muscle strength, abnormal MU firing behavior, and degeneration of the central nervous system [11,26]. These previous findings are in accordance with the results of this study showing that compared with the less-affected side, the more-affected side exhibited less muscle strength, larger abnormalities in MU firing behavior, and more severe degeneration of the central lesion.

The MU firing rate and recruitment threshold line describes an “operating point” of the motor neuron pool that shifts in response to excitation, and the relationship between the MU firing rate and recruitment threshold showed a negative correlation in the vastus lateralis muscle for healthy control subjects [27]. Our results on the less-affected side were consistent with a neural control scheme called “onion skin”. In this scheme, early recruited MUs have a higher MU firing rate than later recruited MUs, and early recruited MUs maintain a higher MU firing rate than later recruited MUs [27,28]. However, the more-affected side did not show a correlation between the recruitment threshold and mean MU firing rate during the submaximal ramp-up contraction. A previous study reported that people with PD showed a possible shift in the MU population to a lower recruitment threshold than healthy control subjects. Furthermore, our previous report showed that there was a significant correlation between the mean MU firing rate and recruitment threshold on the less-affected side but not on the more-affected side [11]. MU firing behavior is primarily determined by the excitatory input of synapses from the corticospinal tract to the motor neuron pool [29]. PD is a degeneration of the substantia nigra compacta, and as the disease progresses, degeneration of cells in the basal ganglia, thalamus, and spinal cord is observed [30,31]. Because motor neurons receive several inputs from the basal ganglia, thalamus, and sensory afferents, degeneration of these regions in people with PD may disrupt the balance of excitatory to motor neurons. In this study, the degree of neurodegeneration coincided with abnormalities in MU firing behavior. These results suggest that central nervous system degeneration can be predicted from muscle, which is a peripheral effector.

The progression of neurodegeneration leads to the worsening of physical symptoms, and in this study, we found a significant correlation between SBR_Bolt_, which reflects the activity of dopaminergic transporters and the integrity of dopaminergic pathways, and UPDRS Part III scores, which reflect physical function (Figure 3D). This finding is in accordance with those of previous studies showing a correlation between the clinical symptoms and time course of progressive PD [3,32]. Importantly, we found that SBR_Bolt_ was associated with the mean MU firing rate, CV of the mean MU firing rate, cross-correlation function, and linear regression slope of the mean MU firing rate and recruitment threshold (Figure 4). Previous studies have indicated that abnormalities in MU firing behavior in people with PD are due to central nervous system degeneration [11,33], but no studies have directly examined the degree of central nervous system degeneration and abnormalities in MU activity. The lower value of the SBR_Bolt_, the greater degeneration of dopaminergic neurons [25]. Furthermore, we calculated cutoff values of the mean MU firing rate, CV of the mean MU firing rate, cross-correlation function, and linear regression slope to predict the severity of degeneration of lesions (SBR > 4.5 or SBR < 4.5). Since this study included only people with PD, there were few subjects with SBR > 4.5, but the AUC of each ROC curve was high (Figure 5), suggesting that abnormalities in MU firing behavior may be used to predict central nervous system degeneration.

Our study has several limitations. First, this study was performed at a single center and small sample size (14 females with PD). This study clearly shows the association between central lesions and MU firing behavior in people with PD. However, the study results should be interpreted with caution because of the effect of the small sample size on the correlation coefficient results. Second, we recruited only females with PD. Therefore, our results cannot be generalized to males with PD. Furthermore, since only people with PD were included in this study, we were not able to obtain data on subjects without neurodegeneration (SBR > 4.5). To improve the accuracy of the cutoff values for abnormal MU firing behavior, we need to recruit healthy subjects and subjects without neurodegeneration. Future studies (e.g., multicenter studies (including healthy subjects) and studies analyzing the influence of sex on the association of central lesions and MU firing behavior) are needed to elucidate the association between MU firing behavior and central lesions in people with PD.

## 5. Conclusions

In summary, we analyzed the association between neurodegeneration and MU firing behavior in people with PD. In this study, there was a significant correlation between neurodegeneration severity and MU firing behavior (e.g., mean MU firing rate, CV of the mean MU firing rate, cross-correlation, and linear regression slope) in people with PD. These findings suggest that abnormalities in MU activity may be used to detect central nervous system degeneration.

## Figures and Tables

**Table 1:** Characteristics of participants.

Variables	Females with Parkinson’s Disease
Age, years	72.6 ± 7.2
Height, cm	157.1 ± 10.2
Weight, kg	58.3 ± 6.8
Disease duration, year	3.5 ± 2.1
MMSE	26.6 ± 1.4
UPDRS Part III	9.9 ± 9.1
L-dopa, mg/day	300 (100–300)
Knee extensor muscle strength, Nm More-/Less-affected side	54.3 ± 9.9/72.9 ± 20.5 *

Data are presented as the mean ± SD or median (min–max). MMSE, Mini-Mental State Examination; UPDRS, Unified Parkinson’s Disease Rating Scale. * *p* < 0.05, compared with the more-affected side.

## Data Availability

Not applicable.
